# Compound musk injection in the treatment of ischemic stroke: A network analysis of the mechanism of action

**DOI:** 10.1097/MD.0000000000036179

**Published:** 2023-11-24

**Authors:** Xiaoqing Li, Hua Yang, Jianjie Cheng, Hairong Zhao, Ya Yan, Qian Wang, Dexiao Wang, Guangming Wang

**Affiliations:** a The First Affiliated Hospital of Dali University, Dali, Yunnan Province, China; b College of Pharmacy, Dali University, Dali, Yunnan Province, China.

**Keywords:** cell experiment, compound musk injection, ischemic stroke, molecular docking, network analysis

## Abstract

**Background:**

Ischemic stroke (IS) is affected by a wide range of factors and has certain treatment limitations. Studies have reported that compound musk injection (CMI) is effective in the treatment of IS, however, its mechanism of action is still unclear.

**Methods:**

The main active ingredients in CMI were retrieved from HERB, TCMSP and BATMAN databases, and the relevant targets were predicted by Swiss Target Prediction platform. MalaCards, OMIM, DrugBank, DisGeNET, Genecards and TTD databases were used to obtain the genes related to IS. The intersection of drugs and disease targets was used to construct protein–protein interaction networks, and gene ontology (GO) enrichment analysis and Kyoto Encyclopedia of Genes and Genomes (KEGG) enrichment analysis were performed. AutoDock Vina software was used for molecular docking, and cell experiments were conducted to verify the results. Reverse transcription-polymerase chain reaction (RT-PCR) was used to detect the expression level of relative mRNA in cells.

**Results:**

Network analysis and molecular docking results showed that the key targets of CMI in the treatment of IS were SRC, TP53, PIK3R1, MAPK3, PIK3CA, MAPK1, etc. KEGG pathway enrichment analysis mainly involved PI3K/Akt signaling pathway, Rap1 signaling pathway and MAPK signaling pathway. The molecular docking results all showed that the key ingredients were strong binding activity with the key targets. The quantitative RT-PCR results indicated that CMI may increase the expression of PIK3CA, MAPK3 mRNA and decrease the expression of SRC mRNA.

**Conclusions:**

CMI can treat IS by regulating pathways and targets related to inflammatory response and apoptosis in a multi-component manner.

## 1. Introduction

Stroke is an acute cerebrovascular disease, including ischemic and hemorrhagic stroke, of which ischemic stroke (IS) accounts for more than 80% of all strokes.^[1]^ IS known as “cerebral infarction” in modern medicine, is a kind of irreversible damage or necrosis of local brain tissue caused by ischemia and hypoxia due to local blood supply disorders caused by a variety of factors. Clinical manifestations included sudden fainting, unconsciousness, slanting tongue, poor language, hemiplegia, etc. IS has a high mortality and disability rate, which brings huge psychological and economic burden to patients. Although thrombolysis or mechanical thrombectomy can improve the condition of patients to a certain extent, due to the limitation of treatment time window and the high risk of hemorrhagic metastasis, only 2%–10% of patients in the world can benefit from it, and 1.2%–3.8% of patients in China can benefit from it.^[2,3]^ There are certain limitations in treatment, which causes the occurrence of alternative and complementary therapy. Therefore, it is critical to seek more effective measures to prevent and treat stroke.

With the continuous improvement of the level of traditional Chinese medicine, a series of Chinese patent medicines for the treatment of IS have been developed through continuous optimization and reform of important prescriptions in clinical practice. Compound Musk injection (CMI) is a kind of medicine widely used in clinical medicine, which includes 6 Chinese herbs: Acori tatarinowii rhizoma, musk, Borneol, Radix Curcumae, Pogostemon cablin and menthol. It is mainly used for stroke coma caused by internal occlusion of phlegm and heat.^[4]^ In modern clinical application, CMI can treat acute cerebral infarction, cerebral hemorrhage, severe craniocerebral injury, pneumonia toxic encephalopathy, vertebrobasilar ischemic vertigo, etc.^[5]^ Musk volatile components can improve motor function after cerebral ischemia by up-regulating the relative levels of AKT1, PI3KA and VEGFA through HIF1A pathway.^[6]^ Studies have shown that borneol can inhibit cell death by inhibiting calcium overload and ferroptosis,^[7,8]^ and can also alleviate brain edema by changing the permeability of the blood-brain barrier.^[9]^ Radix Curcumae, one of the core drugs for the treatment of stroke, inhibits the activation of NLRP3 inflammasome through the NF-κB pathway, thereby reducing the expression levels of pro-inflammatory cytokines IL-1β, IL-6, and TNF-α.^[10]^ Moreover, it may play a protective role in neurons by inhibiting PRV virus-induced neuronal damage through BDNF/Trk B pathway_._^[11]^ Alpha-asarone in acorus tatarinowii can inhibit neuronal calcium overload, regulate glutamate receptors, maintain glutamate concentration, and eliminate glutamate excitability and neurotoxicity.^[12]^ Flavonoids are composed of a group of phenylpropanoids, which can be divided into 12 subclasses according to the degree of oxidation of heterocycles: chalcones, astragalus, flavonoids, flavanones, flavones, isoflavones, phlobaphenes, dihydroflavonols, flavonols, colorless anthocyanins, proanthocyanidins, and anthocyanins.^[13,14]^ They show a wide range of health benefits, including antibacterial, anticancer, anti-osteoporosis and antiviral activities.^[15]^ However, the pharmacological effects of CMI in the treatment of IS are not fully understood.

Network pharmacology is helpful to reveal the mechanism of action of drugs, and the integration strategy of multi-direction pharmacology is helpful to expand the available target space of drugs, increase the success rate of clinical trials, and reduce the cost of finding new drugs.^[16]^ We constructed a network of drug and disease targets through network analysis, identifying potential mechanisms and key targets. Molecular docking technology was used to simulate the interaction of ligand-receptor proteins, and 6 genes with the highest correlation with the disease were screened.^[17]^ RT-PCR was used to detect the mRNA expression levels of the 3 genes. In order to provide a theoretical basis for the subsequent research of CMI.

## 2. Materials and methods

### 2.1. Component screening and target prediction of CMI

Traditional Chinese medicine systems pharmacology database and analysis platform^[18]^ (http://old.tcmsp-e.com/tcmsp.php, TCMSP), Bioinformatics Analysis tool of Molecular mechanism of traditional Chinese medicine (http://bionet.ncpsb.org.cn/batman-tcm/, BATMAN) and herbal database (http://herb.ac.cn/, HERB) were used to search the active components of CMI.

The TCMSP database screened active ingredients based on drug similarity (DL) ≥ 0.18 and oral bioavailability ≥ 30%.^[19]^ Active ingredients were obtained by setting “Score cutof” at 20 and *P* value at .05 based on the predicted score of drug-target interaction prediction during the BATMAN database search.^[20]^ Because the algorithms in each database are different and the names of intellectual chemistry are different, the PubChem database (https://pubchem.ncbi.nlm.nih.gov/) retrieval of each component of the Canonical SMILES number or a 3D structure, and its input Swiss Target Prediction (http://swisstargetprediction.ch/) platform predict related targets.^[21]^ The target was further standardized in UniProtKB database (https://www.uniprot.org/).

### 2.2. Collection of potential therapeutic targets for IS

Using “ischemic stroke” as the key word, the genes related to IS were collected from the following 6 disease-related databases: OMIM (https://www.omim.org/), DisGeNET (http://www.disgenet.org), MalaCards (https://www.malacards.org/pages/info), Genecards (https://www.genecards.org), DrugBank (https://www.drugbank.ca/) and TTD (https://db.idrblab.net/ttd/).^[22]^ The intersection of drug targets and disease targets was selected as the potential therapeutic targets of CMI in the treatment of IS. Targets for drugs and diseases import Venny website (https://bioinfogp.cnb.csic.es/tools/venny/) to get Wayne Figure 2.1.

**Figure 1.**
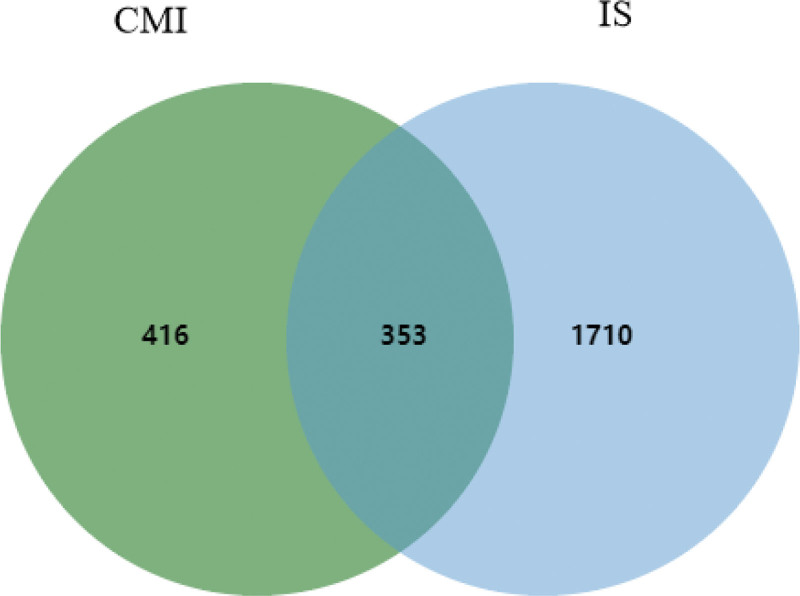
Venn diagram of CMI and IS common targets. CMI = compound musk injection; IS = ischemic stroke.

### 2.3. Construction and analysis of protein–protein interaction networks

The intersection targets were imported into the STRING database (https://www.string-db.org/),^[23]^ and the species was selected as “Homo sapiens,” the minimum required interaction score was set as “Combined score > 0.4,” and the nodes with hidden disconnections were set. Cytoscape software (3.9.1) was used to perform topological analysis and construct the PPI network of key therapeutic targets. The potential therapeutic target genes were ranked according to the degree value, and the targets and active ingredients with higher degree values were screened.^[24]^

### 2.4. Gene ontology and kyoto encyclopedia of genes and genomes analyses

The 353 intersection genes were entered into the Metascape (Http://metascape.org)^[25]^ database for Gene ontology (GO) functional enrichment analysis and Kyoto encyclopedia of genes and genomes (KEGG) pathway enrichment analysis. A threshold of *P* < .05 was used to screen the enrichment results with significant differences, and bar charts and bubble charts were made according to the enrichment results.

### 2.5. Molecular docking

The most important 6 key targets and active ingredients were first selected. The 2D structures of active ingredients were downloaded from PubChem database and imported into Chem Bio 3D Ultra 14.0.0.117 software to obtain the 3D structures, which were saved as mol2 file format and converted to pdbqt file format in AutoDockTools 1.5.6 software. The 3D structure of the protein crystal of the target gene was downloaded from PDB (https://www.rcsb.org/) database and imported into PyMOL 2.5.2 software to remove water molecules and small molecule ligands, and added hydrogen atoms in AutoDockTools 1.5.7 software, and converted to pdbqt file format. Molecular docking was performed using AutoDock Vina (v.1.1.2).^[26]^ Based on the evaluation index of binding energy, it is generally believed that the binding energy < 0.0 kcal/mol indicates that the ligand has binding activity with the target, and the lower the binding energy, the more stable the binding. The docking results were visualized by PyMOL 2.5.2 software.

### 2.6. Cell experiment

#### 2.6.1. Grouping and the construction of OGD/R model.

The digested and resuspended HT-22 cells were randomly divided into Control group (Control), model group (OGD/R), CMI high, medium and low concentration groups (drug content: 0.01%, 0.001%, 0.0001%). The cell concentration was adjusted to 80,000 cells/mL and seeded in 96-well plates with 100uL of cell suspension per well. After being cultured in the cell incubator for 24 hours, the Control group and OGD group were treated with 10% fetal bovine serum (FBS) high-glucose DMEM medium, and the drug group was treated with an equal volume of CMI (drug content: 0.01%, 0.001%, 0.0001%) + 10% FBS high-glucose DMEM medium for 8 hours, respectively. After the pretreatment, the Control group was replaced with 10% FBS high-glucose DMEM medium, the OGD/R group was replaced with serum-free and sugar-free DMEM medium, and the drug group was replaced with equal volume of CMI (drug-containing percentage: 0.01%, 0.001%, 0.0001%) + serum-free and sugar-free DMEM medium. The Control group was put back into the normal cell incubator at 37°C, 5% CO_2_, and the other groups were put into the 3-gas incubator at 37°C, 5% CO_2_, 1% O_2_, 94% N_2_ for oxygen-glucose deprivation (OGD/R) for 12 hours to prepare the OGD/R model. After oxygen-glucose deprivation, the cells in each group were replaced by high-glucose DMEM medium containing 10% FBS, and the cells in the drug group were added with the corresponding concentration of Compound Musk Injection, and cultured in the normal incubator for 12 hours.

#### 2.6.2. Cell viability of HT-22 cells was detected by CCK8 assay.

Serum-free high-glucose DMEM medium was mixed with CCK8 test solution at 10:1, added to the cells (in the dark), 100 μL per well, and returned to the incubator for further treatment for 2 hours. At the end of the treatment, the Optical Density (OD) value of each well was measured at the wavelength of 490 nm by microplate reader. The cell growth survival rate was calculated according to the OD value and cell survival rate formula. Cell survival rate (%) = (OD value of experimental group − OD value of blank group) / (OD value of control group − OD value of blank group) × 100 %.

#### 2.6.3. Quantitative RT-PCR.

HT-22 cells treated with 2.6.1 were collected and total RNA was extracted using TRIzol kit. Cells were first resuspended with 1 mL TRIzol test, transferred to 1.5 mL enzyme-free EP tubes, and allowed to stand for 5 minutes at room temperature. Then 200 μL chloroform was added to each centrifuge tube and centrifuged at 10,000 rpm and 4°C for 10 minutes. Finally, 0.5 mL of isopropanol was added to the EP tube containing total RNA, an appropriate amount of 75% ethanol was added to clean the isopropanol by centrifugation, and 100 μL of DEPC water was added to dissolve the RNA precipitate.

The primer sequences were reviewed in the NCBI database as shown in Table 1 and synthesized by Sangon Bioengineering (Shanghai) Co., LTD. Quantitative RT-PCR reaction system was established: 5 μL 2x Realab Green PCR Fast mixture, 0.75 μL forward and reverse primers, and 4.25 μL cDNA. Reaction conditions: pre-deformation at 95°C for 30 seconds; Denaturation at 95°C for 10 seconds; 40 cycles of annealing/extension at 60°C for 30 seconds were performed for relative quantification according to the 2^−∆∆Ct^ method.

**Table 1:** Primer sequence.

Primer name	Primer orientation	Sequence (5’-3’)	Product length
SRC	F	5’-TCTGAACCAAGGCAGCATCT-3’	108
SRC	R	5’-TGGCCTAAAGACCCTGTTGC-3’	108
MAPK3	F	5’-CACTGGCTTTCTGACGGAGT-3’	85
MAPK3	R	5’-GGATTTGGTGTAGCCCTTGGA-3’	85
PIK3CA	F	5’-GAGAAGCCGGAGCGGCA-3’	117
PIK3CA	R	5’-GTTCACCCGAAGATGGTCGT-3’	117
beta-Actin	F	5’-CCTCACTGTCCACCTTCCA-3’	120
beta-Actin	R	5’-GGGTGTAAAACGCAGCTCA-3’	120

## 3. Results

### 3.1. Screening of potentially therapeutic targets of CMI for IS

After eliminating the duplicate items, 136 active ingredients were obtained, including 20 for musk, 1 for menthol, 54 for radix cureumae, 39 for patchouli, 37 for tatarinoa, and 18 for borneol (Table 2). The active ingredients of CMI were input into SwissTarget database, and 769 targets were obtained after deleting duplicate values. 2063 genes associated with IS were retrieved from OMIM, DisGeNET, MalaCards, Genecards, DrugBank and TTD databases. By taking the intersection of disease and drug targets, 353 common targets were obtained (Fig. 1).

**Table 2:** Information of the candidate active ingredients of CMI.

Herb	Active ingredients
Musk	Estradiol; N-Nornuciferine; 17-Beta-estradiol; 3, 5-Dihydroxybenzoic acid; 3-AlPCa-hydroxy-5alPCa-androstan-17-one; 3-Beta-hydroxy-5alPCa-androstan-17-one; 3-Methylcyclotridecan-1-one; 5-cis-Cyclopentadecen-1-one; 5-cis-Cyclotetradecen-1-one; AlPCa-estradiol; Androst-4-ene-3, 17-dione; Androsterone; Cholesterol; Cyclotetradecan-1-one; Decamine; Morin; Muscone; Muscopyridine; Normuscone; Testosterone
Radix Curcumae	CamPCor; PCellatin; CamPCerenol; Turricolol E; Aniline; Tomatidenol; Curdione; CaryoPCyllene; Cibarian; beta-sitosterol; sitosterol; Curcumenolactone C; Zedoalactone A; naringenin; Bicyclo(4.1.0)heptan-3-one, 4-(1-hydroxy-1-methylethyl)-1-methyl-7-(3-oxobutyl)-, (1S,4S,6R,7R)-; Curcarabranol B; D-CamPCor; Cirsilineol; (1aR,4aS,7R,7aR,7bR)-1,1,7-Trimethyl-4-methylidenedecahydro-1H-cyclopropa(e)azulen-7-ol; Procurcumenol; Curlone; 1,7-DiPCenyl-4,6-heptadien-3-one; (-)-Limonene; (+)-alPCa-Terpineol;(Z)-3-(4-hydroxyPCenyl)prop-2-enethioic S-acid; Cinnamaldehyde; (2R)-5,7-dihydroxy-2-(4-hydroxyPCenyl)-2,3-dihydro-4H-chromen-4-one; Caffeic acid; Ethyl ferulate; L-Borneol; cis-p-Coumaric acid; cis-Caffeic acid; (Z)-p-Methoxycinnamic acid; (+)-Terpinen-4-ol; Quercetin; Scopoletin; Capillarisin; IsocaryoPCyllene; Isorhamnetin; Bisdemethoxycurcumin; NCGC00385952-01_C15H26O_1,7-Dimethyl-7-(4-methyl-3-penten-1-yl)bicyclo[2.2.1]heptan-2-ol; cis-Cinnamic acid; Demethoxycurcumin; Zerumbone; (-)-Isoborneol; (E)-2-epi-beta-caryoPCyllene; (Z)-caryoPCyllene; 10-epi-gamma-Eudesmol; Germacrene a; L-Octanoylcarnitine; Curcumalactone; CID 44630107; Salicylate; (3R,10S)-6,10-dimethyl-3-propan-2-ylcyclodec-6-ene-1,4-dione
Acori Tatarinowii Rhizoma	Beta-Asarone; AlPCa-Asarone; Thymol; Nonanoic Acid; Eugenol; Octanoic Acid; Myristic Acid; P-Methoxycinnamic Acid; Asarone; 8-Isopentenyl-kaempferol; Cycloartenol; N,N-Diethylbenzylamine; Heterotropan; D-CamPCor; (Z)-3-(4-hydroxyPCenyl)prop-2-enethioic S-acid; Methylisoeugenol; Caffeic acid; 4-Methoxycinnamic acid; L-Borneol; Isoeugenyl methyl ether; (+)-Terpinen-4-ol; Apigenin; Kaempferol; IsocaryoPCyllene; Isoelemicin; 8-Prenylkaempferol; (Z)-caryoPCyllene; 2H-Cyclopropa[a]naPCthalen-2-one, 1,1a,4,5,6,7,7a,7b-octahydro-1,1,7,7a-tetramethyl-, (1aalPCa,7alPCa,7aalPCa,7balPCa)-; (3R,6E)-nerolidol; L-Octanoylcarnitine; alPCa-Cadinene; 1,4,4-Trimethyl-8-methylene-1,5-cycloundecadiene; 1-(4-Hydroxy-2-methoxyPCenyl)-3-(4-hydroxyPCenyl)prop-2-en-1-one; (2R,3R,4R,5S)-2,5-Bis-(3,4-dimethoxy-PCenyl)-3,4-dimethyl-tetrahydro-furan; 3,4-Dihydroxybenzoate; (1aS,4aS,7S,7aR,7bS)-1,1,7-trimethyl-4-methylidene-1a,2,3,4a,5,6,7a,7b-octahydrocyclopropa[h]azulen-7-ol; (1S,2S,7S)-3,3,5,8,10,10-hexamethoxy-7,11-bis(prop-2-enyl)tricyclo[6.2.2.02,7]dodeca-5,11-diene-4,9-dione
Pogostemon Cablin	Rhamnocitrin; Rhamnetin; Pogostol; Valepotriate; Dibutyl PCthalate; Flopropione; Diisobutyl PCthalate; N-PCenyl-1-naPCthylamine; 5-Methyl-2-hexanone; Nonyl acetate; Widdrol; (1aR,4aS,7R,7aR,7bR)-1,1,7-Trimethyl-4-methylidenedecahydro-1H-cyclopropa(e)azulen-7-ol; Limonin; 5-Hydroxy-7-methoxy-2-(4-methoxyPCenyl)-2,3-dihydrochromen-4-one; (-)-Perillyl alcohol; Cedr-8-en-13-ol; Anethole; Magnograndiolide; Retusin; 3,5-Dihydroxy-7-methoxy-2-(4-methoxyPCenyl)-4H-chromen-4-one; cis-Cinnamaldehyde; 2H-Cyclopropa[a]naPCthalen-2-one, 1,1a,4,5,6,7,7a,7b-octahydro-1,1,7,7a-tetramethyl-, (1aalPCa,7alPCa,7aalPCa,7balPCa)-; (3R,6E)-nerolidol; (2E)-3-[(3aS,5aR,6R,7R,9aR,9bR,10aS)-3-(Furan-3-yl)-7-(2-hydroxypropan-2-yl)-3a,6,9a-trimethyl-1,9-dioxododecahydronaPCtho[2,1-c]oxireno[d]pyran-6-yl]prop-2-enoic acid;(2E)-3-(4-hydroxy-3-methoxyPCenyl)prop-2-enoate; Genkwanin;quercetin 7-O-β-D-glucoside; Acanthoside B; Pachypodol; Eugenol; Patchoulenone; Perilla Ketone; Cinnamaldehyde; Cinnamic Acid; Apigenin; Patchouli Alcohol; Quercetin; Irisolidone; Ombuin
Borneol	[(1S)-endo]-(-)-Borneol; CamPCor; Dryobalanone; Disenecionyl Cis-Khellactone; AlPCitolic Acid; Asiatic Acid; D-Borneol; bronyl acetate; dipterocarpol; Paeonol; (+)-alPCa-Terpineol; (-)-CamPCor; Isosafrole; Baicalein; (-)-Isoborneol; (+)-Borneol; CID 44630107; [(1R,3S,6R,8R)-8-hydroxy-3-methyl-5-oxo-2,9-dioxatricyclo[4.3.1.03,8]decan-10-yl]methyl benzoate
Menthol	menthol

### 3.2. Protein–protein interaction network analysis

**Figure 2.**
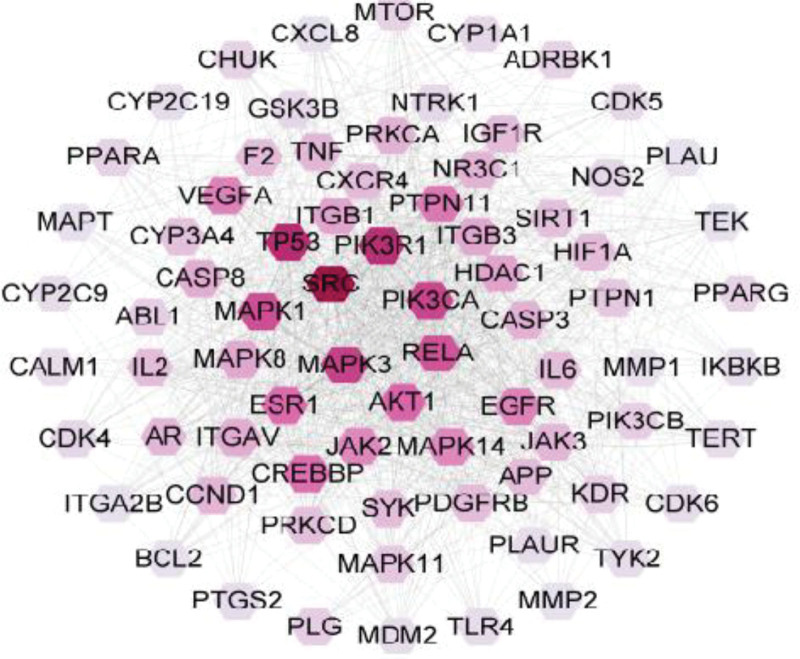
Protein–protein interaction network of key targets.

The 353 potential therapeutic target genes were imported into the STRING database with confidence > 0.4. Then Cytoscape 3.9.1 software was used to perform topological analysis and construct PPI network. Nodes with darker colors in the network represent larger degree values. A critical target network consisting of 76 nodes and 1028 edges was selected based on the double median of the calculated Degree value (Fig. 2). The top 10 targets included SRC, TP53, PIK3R1, MAPK3, PIK3CA, MAPK1, RELA, AKT1, ESR1, and CREBBP (Table 3), which are important targets of CMI in the treatment of IS.

**Table 3:** Topological parameters of the top 20 potential targets.

Gene	Degree	Betweenness centrality	Closeness centrality
SRC	114	0.123725382	0.430577223
TP53	94	0.167040116	0.438095238
PIK3R1	92	0.040949213	0.392603129
MAPK3	84	0.046200836	0.410714286
PIK3CA	82	0.033117323	0.399421129
MAPK1	78	0.040145784	0.408888889
RELA	76	0.074611633	0.426584235
AKT1	68	0.042537451	0.404099561
ESR1	66	0.105096604	0.417549168
CREBBP	64	0.091422084	0.404692082
PTPN11	60	0.012160304	0.37704918
VEGFA	56	0.028070465	0.378600823
EGFR	56	0.033644398	0.393723252
MAPK14	54	0.038091535	0.397122302
JAK2	54	0.024503912	0.37398374
HDAC1	44	0.030346039	0.37449118
ITGB1	44	0.021372074	0.357050453
ITGB3	42	0.00594634	0.34543179
MAPK8	42	0.017894412	0.371467026
ITGAV	40	0.005583193	0.340740741

### 3.3. Analysis of active ingredients-targets network

**Figure 3.**
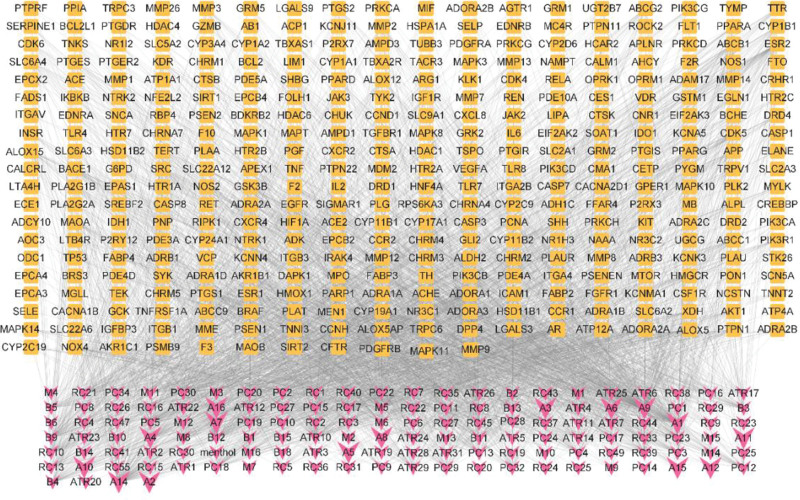
The active ingredients-targets network. Pink represents active ingredients, and Orange represents the potential targets.

In order to establish the correlation between the active ingredients of CMI and potential therapeutic targets, Cytoscape 3.9.1 software was used to obtain the “active ingredient-target” interaction network (Fig. 3, Supplementary Table A1, http://links.lww.com/MD/K767), which included 489 nodes (136 components, 353 targets) and 3039 edges. The degree value reflects the number of nodes directly linked to the compound and is used to assess the importance of the compound in the network. The top 20 active ingredients were screened by topological analysis (Table 4). Apigenin, Quercetin, L-Octanoylcarnitine, Morin, Kaempferol and Asiatic have high acid values and can act on multiple disease targets, which are important active ingredients of CMI in the treatment of IS.

**Table 4:** Top 20 key ingredient information.

Ingredient name	Herbs	Degree	Betweenness centrality
Apigenin	Acori Tatarinowii Rhizoma,Pogostemonis cablin	153	0.013842217
Quercetin	Acori Tatarinowii Rhizoma,Pogostemonis cablin	150	0.009898314
L-Octanoylcarnitine	Curcumae Radix,Pogostemonis cablin	122	0.062591469
Morin	Musk	103	0.013845366
Kaempferol	Acori Tatarinowii Rhizoma	98	0.01011157
asiatic acid	Borneol	75	0.022091965
Irisolidone	Pogostemonis cablin	63	0.023150307
bronyl acetate	Borneol	62	0.052740058
Bisdemethoxycurcumin	Curcumae Radix	61	0.017581846
1,7-DiPCenyl-4,6-heptadien-3-one	Curcumae Radix	59	0.061654747
naringenin	Curcumae Radix	59	0.046966705
Baicalein	Borneol	57	0.028876709
Disenecionyl Cis-Khellactone	Borneol	57	0.06050997
Dibutyl PCthalate	Pogostemonis cablin	55	0.05918266
Eugenol	Acori Tatarinowii Rhizoma,Pogostemonis cablin	54	0.009929236
Ombuin	Pogostemonis cablin	54	0.383956386
3,5-Dihydroxy-7-methoxy-2-(4-methoxyPCenyl)-4H-chromen-4-one	Pogostemonis cablin	53	0.382763975
Retusin	Pogostemonis cablin	53	0.383359253
1-(4-Hydroxy-2-methoxyPCenyl)-3-(4-hydroxyPCenyl)prop-2-en-1-one	Acori Tatarinowii Rhizoma	52	0.379230769
5-Hydroxy-7-methoxy-2-(4-methoxyPCenyl)-2,3-dih ydrochromen-4-one	Pogostemonis cablin	52	0.380989181

### 3.4. GO enrichment analysis

**Figure 4.**
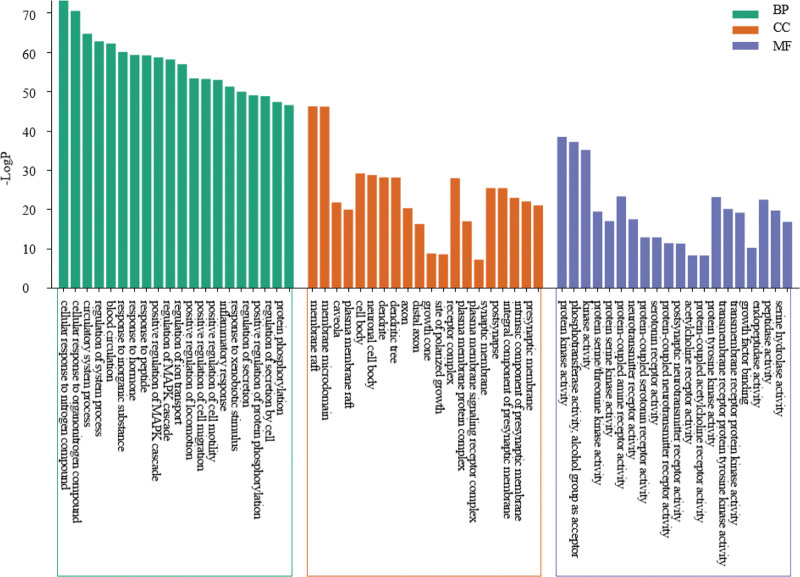
The GO enrichment analysis. GO = gene ontology, KEGG = Kyoto Encyclopedia of Genes and Genomes.

GO functional enrichment analysis of 353 common targets was performed through the Matesape database, and a total of 2851 items were enriched for biological process (BP), 183 items for cell composition (CC), and 375 items for molecular function. Ranked by *P* value, the top 20 items were selected for visual presentation (Fig. 4). Among them, BP is mainly involved in cellular response to nitrogen compound, positive regulation of MAPK cascade, blood circulation, inflammatory response and transmembrane receptor protein tyrosine kinase signaling pathway and so on. CC mainly involves in membrane raft, membrane microdomain, neuronal cell body, synaptic membrane, dendrite, plasma membrane protein complex and so on. Molecular function is mainly involved in protein kinase activity, protein serine/threonine/tyrosine kinase activity, G protein-coupled peptide receptor activity, serotonin receptor activity, neurotransmitter receptor activity and acetylcholine receptor activity and so on.

### 3.5. KEGG pathway enrichment analysis

**Figure 5.**
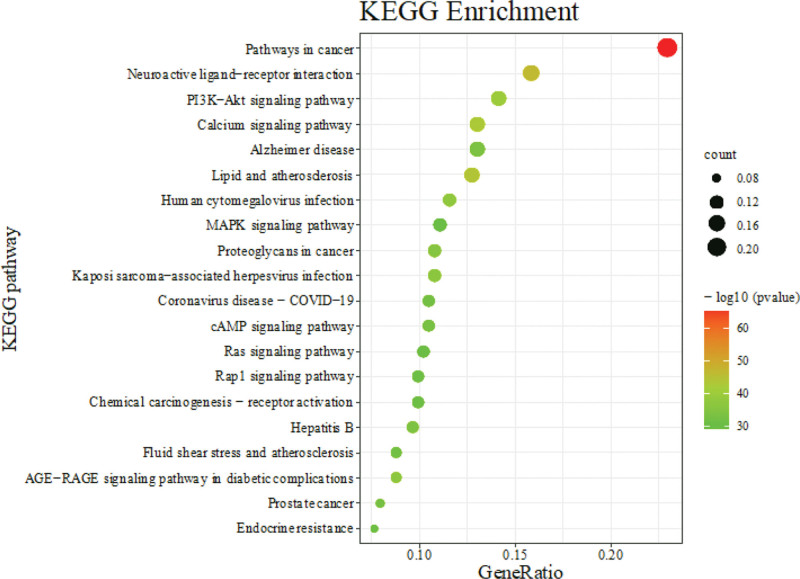
The KEGG enrichment analysis. KEGG = Kyoto Encyclopedia of Genes and Genomes.

A total of 353 targets of CMI in the treatment of stroke were entered into the Matescape database for KEGG pathway enrichment analysis, and 224 pathways were enriched. They were mainly involved in the Pathways in cancer, Neuroactive ligand-receptor interaction, Lipid and atherosclerosis, PI3K-Akt signaling pathway, Calcium signaling pathway, Human cytomegalovirus infection, AGE-RAGE signaling pathway in diabetic complications, cAMP signaling pathway, Coronavirus disease-COVID-19, Rap1 signaling pathway, Endocrine resistance, MAPK signaling pathway. n order of *P* value, we selected the top 20 signaling pathways for bubble plots (Fig. 5).

### 3.6. Docking results analysis

**Figure 6.**
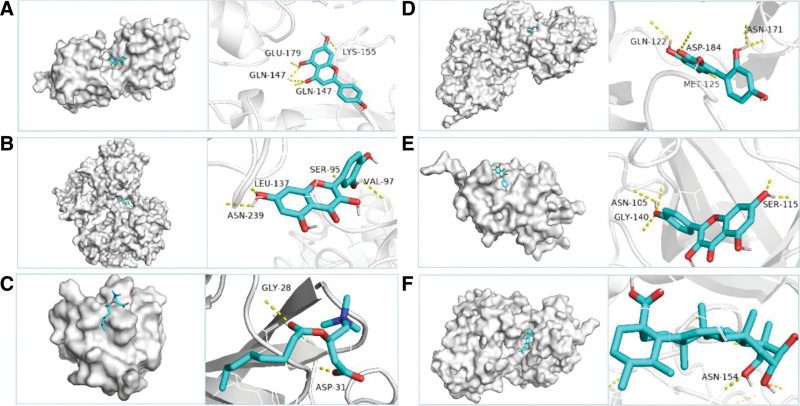
Molecular docking model diagram of 6 key components and 6 key targets. Notes: (A) SRC-apigenin, (B) TP53-quercetin, (C) PIK3R1- L-Octanoylcarnitine, (D) MAPK3-morin, (E) PIK3CA-kaempferol, (F) MAPK1-asiatic acid.

In order to analyze the feasibility of CMI in the treatment of IS, we performed molecular docking. The binding energy is an important parameter, and the binding strength is inversely proportional to the size of the binding energy. When the binding energy is less than − 5.0 kcal/mol, it indicates that the docking model has good binding activity.^[27]^ Apigenin, Quercetin, L-Octanoylcarnitine, Morin, Kaempferol and Asiatic acid were linked to key targets SRC, TP53, PIK3R1, MAPK3, PIK3CA and MAPK1, respectively. The results showed that all the 6 key components bound to the key targets and had stable docking models (Table 5, Fig. 6).

**Table 5:** Docking results of key active ingredients with key targets (kcal/mol).

Gene	Apigenin	Quercetin	L-Octanoylcarnitine	Morin	Kaempferol	Asiatic acid
SRC	−6.8	−6.9	−5.2	−6.7	−7.2	−7.3
TP53	−6.9	−7.2	−5.1	−7.2	−7.1	−8.3
PIK3R1	−7.0	−7.1	−5.1	−6.3	−6.5	−7.2
MAPK3	−8.7	−9.4	−6.6	−9.1	−8.9	−8.6
PIK3CA	−6.2	−6.0	−5.1	−6.1	−6.0	−6.3
MAPK1	−8.5	−8.4	−5.6	−8.4	−8.1	−9.0

### 3.7. Effect of CMI on cell survival rate

**Figure 7.**
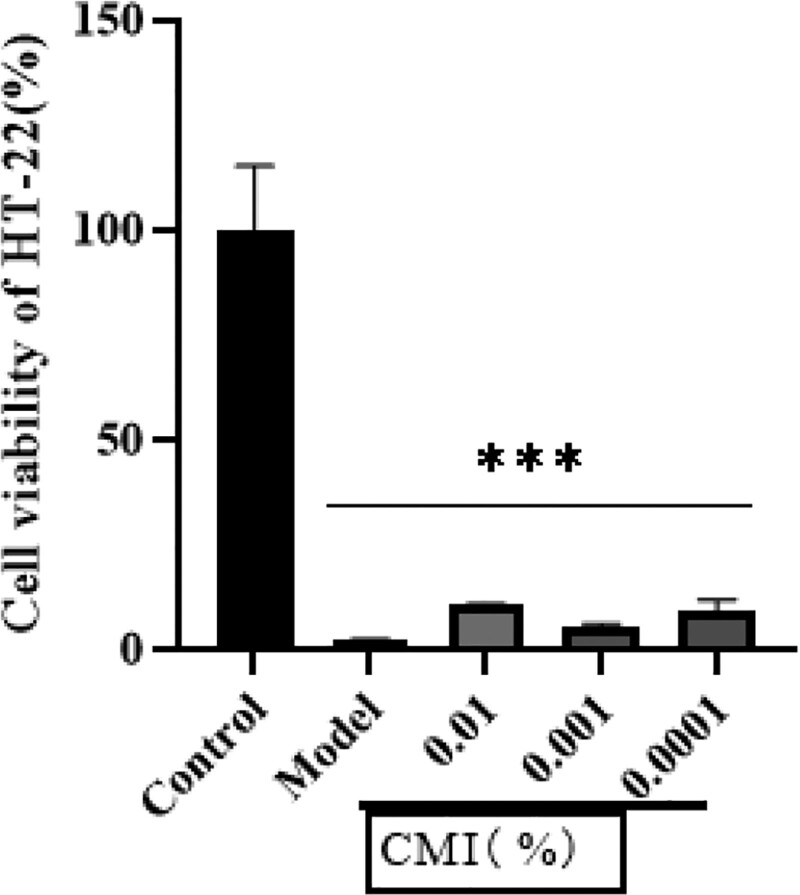
Effect of CMI on cell survival rate of OGD/R model. Notes: ***P < .01 vs control group. CMI = compound musk injection; OGD/R = oxygen-glucose deprivation/reoxygenation.

Cell experiments showed that the survival rate of HT-22 cells was significantly decreased after oxygen-glucose deprivation/reoxygenation, and the difference was statistically significant compared with the blank group (*P* < .01). Compared with the model group, the CMI groups had a tendency to improve the cell survival rate, as shown in Figure 7 (*P* > .05).

### 3.8. Expression of SRC, PIK3CA, MAPK3 mRNA

**Figure 8.**
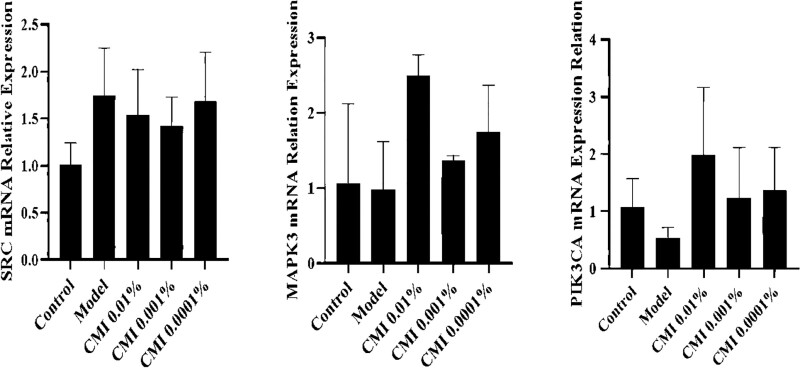
Protein expression of SRC, PIK3CA, and MAPK3.

Compared with the blank group, the mRNA expressions of PIK3CA and MAPK3 in the Model group tended to decrease, while the mRNA expression of SRC tended to increase (*P* > .05). Compared with the Model group, the expression of PIK3CA and MAPK3 mRNA in the CMI group (drug percentage: 0.01%, 0.001%, 0.0001%) tended to increase, and the expression of SRC mRNA tended to decrease (Fig. 8). Therefore, CMI may increase the expression of PIK-3CA and MAPK-3 mRNA and decrease the expression of SRC mRNA.

## 4. Conclusions

IS mainly originates from atherosclerosis, which leads to a series of pathological processes such as energy metabolism disorders, excitatory amino acid toxicity, oxidative/nitrifying stress, inflammatory response, apoptosis, and autophagy in brain tissue. Traditional Chinese medicine can be used in different stages of disease due to its multi-component and multi-target therapeutic characteristics. The network pharmacology method was used to find 136 active ingredients from 6 kinds of traditional Chinese medicine in CMI, and 353 potential therapeutic targets were screened out from the traditional Chinese medicine targets. Key targets such as SRC, TP53, PIK3R1, MAPK3, PIK3CA and MAPK1 were obtained through PPI network. It is closely related to PI3K-Akt signaling pathway and MAPK signaling pathway.

Src is a non-receptor protein tyrosine kinase, which can regulate extracellular signal-regulated kinase in ischemic brain tissue, and then play a protective role in brain.^[28]^ It has been found that salvianolic acid A protects cerebral vascular endothelial cells from ischemia and OGD injury by inhibiting Src signaling pathway.^[29]^ Inhibition of Src expression reduces inflammatory cytokine secretion, oxidative stress, and astrocyte apoptosis.^[30]^ TP53 is an important gene involved in the initiation process of apoptosis and has an important role in regulating cell growth and DNA repair progression.^[31]^ When IS occurs, p53 expression levels are rapidly upregulated, which directly disrupts the permeability of the mitochondrial membrane by enhancing the Bcl-2 family of proapoptotic proteins (PUMA and BAX), thereby damaging cells in the ischemic semidark zone.^[32]^ The MAPK signaling pathway involves can be grouped into 3 main families of intracellular serine/threonine protein kinases that include extracellular signal-regulated kinase (ERK), p38, and c-Jun NH2-terminal kinase (JNK).^[33]^ Activated MAPK, which phosphorylates and activates downstream ERK kinases ERK-1 and ERK-2, ultimately transmits cell proliferation and differentiation signals to the nucleus by regulating the activity of transcription factors.^[34,35]^ MAPK contributes to the maintenance of neural stem cells and can be activated by phosphorylation of threonine and tyrosine residues in response to cerebral ischemia. MAPK phosphorylation can promote neuronal survival in the dentate gyrus region.^[36]^ In the present study, CMI increased MAPK mRNA expression in OGD/R model cells, indicating that CMI could act on MAPK targets. PIK3CA is the catalytic subunit encoding PI3K, and PIK3R1 is the regulatory subunit of phosphatidylinositol-3-kinase (PI3Ks). In the process of stroke, PIK3CA and PIK3R1 are the main genes that promote cell survival and reduce cell apoptosis.^[37,38]^

PI3K-Akt signaling pathway is a key pathway in the pathological mechanism of ischemic brain injury, which mainly regulates cell survival, proliferation, metabolism, neuroscience, movement and cancer progression.^[39,40]^ Studies have demonstrated the neuroprotective effects of Danhong injection in rats with ischemia-reperfusion injury and suggest that these effects on the brain are in part due to activation of the PI3K-Akt signaling pathway.^[41]^ The PI3K inhibitor LY294002 decreased Akt activity, which partially inhibited the protective effect on infarct size reduction, and increased Akt activity correlates with changes in glycogen synthase kinase 3β (GSK-3β) phosphorylation shortly after cerebral ischemia and changes in β-catenin phosphorylation patterns in the ischemic core and penumbra.^[42]^ Fucoidan may protect cerebral ischemia-reperfusion injury by inhibiting MAPK pathway.^[43]^ OXA may play a neuroprotective role in CIRI by inhibiting NF-κB, MAPK/ERK and MAPK/p38 signaling pathways mediated by OX1R, and inhibiting astrocyte apoptosis, activation and proinflammatory cytokine production.^[44]^ Endogenous inhibition of p38 MAPK rescues hippocampal apoptosis, reduces ischemic penumbra, and ameliorates neurobehavioral deficits.^[45]^ cAMP/PKA signaling pathway plays an important role in inhibiting neuronal apoptosis. Once the cell is stimulated, the signaling molecule binds to the receptor on the cell membrane to form a complex, which then activates adenylate cyclase. Eventually cAMP is produced, which enters the nucleus and directly activates RNA polymerase to promote mRNA transcription of the target gene.^[46,47]^ Lidocaine may improve the neural function and inhibit the neuronal apoptosis in CIRI rats by activating cAMP/PKA signaling pathway.^[48]^ It has been found that cAMP can participate in many signaling pathways initiated by neurotransmitters, among which cAMP can directly act on the specific guanylate exchange factor EPAC of Rap1 to induce its activation, and activated EPAC can directly activate the downstream Rap1.^[49]^ Inhibition of Epac pathway by Rap1 inhibitor GGTI298 and Rac1 inhibitor NSC23766 can aggravate blood-brain barrier injury and brain injury, suggesting that activation of Epac/Rap 1 signaling pathway has neuroprotective effect on cerebral ischemia-reperfusion injury.^[50]^

This study lays a foundation for further study on the mechanism of CMI, provides a theoretical basis for further systematic experiments, and provides new ideas for the targeted therapy of IS. However, this study still has some limitations. Due to the limited data in the database and related literature, the therapeutic targets, related pathways and further mechanisms of action predicted in this paper need to be carried out in relevant systematic experimental empirical work.

## 5. Conclusions

In this study, network pharmacological analysis, molecular docking technology and cell experiments were used to explore the mechanism of CMI in the treatment of IS. The results showed that Apigenin, Quercetin, Morin and other components in CMI may act on targets such as SRC, TP53, PIK3R1, MAPK3, PIK3CA, MAPK1 and other targets through PI3K/AKT and MAPK signaling pathways. Thus, it can reduce reperfusion injury, inhibit cell apoptosis and anti-inflammation. It provides some theoretical basis for further determining the mechanism of action of traditional Chinese medicine in the prevention and treatment of IS.

## Author contributions

**Conceptualization:** Xiaoqing Li, Ya Yan, Qian Wang, Dexiao Wang.

**Data curation:** Xiaoqing Li, Hua Yang, Jianjie Cheng, Hairong Zhao, Qian Wang, Dexiao Wang.

**Formal analysis:** Xiaoqing Li, Jianjie Cheng, Hairong Zhao, Qian Wang, Dexiao Wang.

**Methodology:** Xiaoqing Li, Hairong Zhao.

**Project administration:** Guangming Wang.

**Software:** Xiaoqing Li, Qian Wang, Dexiao Wang.

**Supervision:** Guangming Wang, Hua Yang, Jianjie Cheng, Ya Yan.

**Validation:** Hua Yang.

**Visualization:** Xiaoqing Li.

**Writing – original draft:** Xiaoqing Li.

**Writing – review & editing:** Guangming Wang, Hairong Zhao.

## Supplementary Material
