# Supplementary material for: Compound musk injection in the treatment of ischemic stroke: A network analysis of the mechanism of action
Source: Medicine (Baltimore). 2023 Nov 24;102(47):e36179. doi: 10.1097/MD.0000000000036179 (PMC10681625; doi:10.1097/MD.0000000000036179)
Supplement: Supplementary file 1 [file medi-102-e36179-s001.docx]

**Table A1.** Coding list of active ingredients.

| **Herb name** | **Ingredient name** | **Serial number** |
| --- | --- | --- |
| Radix Curcumae；Acori Tatarinowii Rhizoma | L-Octanoylcarnitine | A1 |
| Radix Curcumae；Acori Tatarinowii Rhizoma | (Z)-3-(4-hydroxyPCenyl)prop-2-enethioic S-acid | A10 |
| Radix Curcumae；Pogostemon Cablin | (1aR,4aS,7R,7aR,7bR)-1,1,7-Trimethyl-4-methylidenedecahydro-1H-cyclopropa(e)azulen-7-ol | A11 |
| Radix Curcumae；Borneol | (+)-alPCa-Terpineol | A12 |
| Radix Curcumae；Pogostemon Cablin | Quercetin | A14 |
| Acori Tatarinowii Rhizoma；Pogostemon Cablin | 2H-Cyclopropa[a]naPCthalen-2-one, 1,1a,4,5,6,7,7a,7b-octahydro-1,1,7,7a-tetramethyl-,(1aalPCa,7alPCa,7aalPCa,7balPCa)- | A15 |
| Radix Curcumae；Borneol | (-)-Isoborneol | A16 |
| Radix Curcumae；Acori Tatarinowii Rhizoma | L-Borneol | A2 |
| Radix Curcumae；Acori Tatarinowii Rhizoma | IsocaryoPCyllene | A3 |
| Acori Tatarinowii Rhizoma；Pogostemon Cablin | Eugenol | A4 |
| Radix Curcumae；Acori Tatarinowii Rhizoma | D-CamPCor | A5 |
| Radix Curcumae；Borneol | CID 44630107 | A6 |
| Radix Curcumae；Borneol | CamPCor | A7 |
| Radix Curcumae；Acori Tatarinowii Rhizoma | Caffeic acid | A8 |
| Acori Tatarinowii Rhizoma；Pogostemon Cablin | Apigenin | A9 |
| Acori Tatarinowii Rhizoma | Thymol | ATR1 |
| Acori Tatarinowii Rhizoma | Kaempferol | ATR10 |
| Acori Tatarinowii Rhizoma | Isoeugenyl methyl ether | ATR11 |
| Acori Tatarinowii Rhizoma | Isoelemicin | ATR12 |
| Acori Tatarinowii Rhizoma | Heterotropa | ATR14 |
| Acori Tatarinowii Rhizoma | Cycloartenol | ATR17 |
| Acori Tatarinowii Rhizoma | Beta-Asarone | ATR19 |
| Acori Tatarinowii Rhizoma | P-Methoxycinnamic Acid | ATR2 |
| Acori Tatarinowii Rhizoma | Asarone | ATR20 |
| Acori Tatarinowii Rhizoma | alPCa-Cadinene | ATR22 |
| Acori Tatarinowii Rhizoma | AlPCa-Asarone | ATR23 |
| Acori Tatarinowii Rhizoma | 8-Prenylkaempferol | ATR24 |
| Acori Tatarinowii Rhizoma | 8-Isopentenyl-kaempferol | ATR25 |
| Acori Tatarinowii Rhizoma | 4-Methoxycinnamic acid | ATR26 |
| Acori Tatarinowii Rhizoma | 1,4,4-Trimethyl-8-methylene-1,5-cycloundecadiene | ATR28 |
| Acori Tatarinowii Rhizoma | 1-(4-Hydroxy-2-methoxyPCenyl)-3-(4-hydroxyPCenyl)prop-2-en-1-one | ATR29 |
| Acori Tatarinowii Rhizoma | Octanoic Acid | ATR3 |
| Acori Tatarinowii Rhizoma | (2R,3R,4R,5S)-2,5-Bis-(3,4-dimethoxy-PCenyl)-3,4-dimethyl-tetrahydro-furan | ATR31 |
| Acori Tatarinowii Rhizoma | Nonanoic Acid | ATR4 |
| Acori Tatarinowii Rhizoma | N,N-Diethylbenzylamine | ATR5 |
| Acori Tatarinowii Rhizoma | Myristic Acid | ATR6 |
| Acori Tatarinowii Rhizoma | Methylisoeugenol | ATR7 |
| Borneol | Paeonol | B1 |
| Borneol | (+/-)-Isoborneol | B10 |
| Borneol | Baicalein | B11 |
| Borneol | Asiatic Acid | B12 |
| Borneol | AlPCitolic Acid | B13 |
| Borneol | [(1R,3S,6R,8R)-8-hydroxy-3-methyl-5-oxo-2,9-dioxatricyclo[4.3.1.03,8]decan-10-yl]methyl benzoat | B14 |
| Borneol | (+)-Borneol | B15 |
| Borneol | (-)-CamPCor | B18 |
| Borneol | Isosafrole | B2 |
| Borneol | Dryobalanone | B3 |
| Borneol | Disenecionyl Cis-Khellactone | B4 |
| Borneol | dipterocarpol | B5 |
| Borneol | D-Borneol | B6 |
| Borneol | bronyl acetate | B9 |
| musk | Testosterone | M1 |
| musk | Androst-4-ene-3, 17-dione | M10 |
| musk | AlPCa-estradiol | M11 |
| musk | 5-cis-Cyclotetradecen-1-one | M12 |
| musk | 3-Methylcyclotridecan-1-one | M13 |
| musk | 3-Beta-hydroxy-5alPCa-androstan-17-one | M14 |
| musk | 3-AlPCa-hydroxy-5alPCa-androstan-17-one | M15 |
| musk | 17-Beta-estradiol | M16 |
| musk | Normuscone | M2 |
| musk | N-Nornuciferine | M3 |
| musk | Muscone | M4 |
| musk | Morin | M5 |
| musk | Estradiol | M6 |
| musk | Cyclotetradecan-1-one | M7 |
| musk | Cholesterol | M8 |
| musk | Androsterone | M9 |
| menthol | menthol | menthol |
| Pogostemon Cablin | Widdrol | PC1 |
| Pogostemon Cablin | Patchouli Alcohol | PC10 |
| Pogostemon Cablin | Patchoulenone | PC11 |
| Pogostemon Cablin | Pachypodol | PC12 |
| Pogostemon Cablin | Ombuin | PC13 |
| Pogostemon Cablin | N-PCenyl-1-naPCthylamine | PC14 |
| Pogostemon Cablin | Nonyl acetate | PC15 |
| Pogostemon Cablin | Magnograndiolide | PC16 |
| Pogostemon Cablin | Limonin | PC17 |
| Pogostemon Cablin | Irisolidone | PC18 |
| Pogostemon Cablin | Genkwanin | PC19 |
| Pogostemon Cablin | Valepotriate | PC2 |
| Pogostemon Cablin | Flopropione | PC20 |
| Pogostemon Cablin | Diisobutyl PCthalate | PC22 |
| Pogostemon Cablin | Dibutyl PCthalate | PC23 |
| Pogostemon Cablin | Cinnamic Acid | PC24 |
| Pogostemon Cablin | Cedr-8-en-13-ol | PC25 |
| Pogostemon Cablin | Anethole | PC27 |
| Pogostemon Cablin | Acanthoside B | PC28 |
| Pogostemon Cablin | 5-Hydroxy-7-methoxy-2-(4-methoxyPCenyl)-2,3-dihydrochromen-4-one | PC29 |
| Pogostemon Cablin | Rhamnocitrin | PC3 |
| Pogostemon Cablin | 3,5-Dihydroxy-7-methoxy-2-(4-methoxyPCenyl)-4H-chromen-4-one | PC30 |
| Pogostemon Cablin | (2E)-3-[(3aS,5aR,6R,7R,9aR,9bR,10aS)-3-(Furan-3-yl)-7-(2-hydroxypropan-2-yl)-3a,6,9a-trimethyl-1,9-dioxododecahydronaPCtho[2,1-c]oxireno[d]pyran-6-yl]prop-2-enoic aci | PC32 |
| Pogostemon Cablin | (-)-Perillyl alcohol | PC34 |
| Pogostemon Cablin | Rhamnetin | PC4 |
| Pogostemon Cablin | Retusin | PC5 |
| Pogostemon Cablin | quercetin 7-O-β-D-glucoside | PC6 |
| Pogostemon Cablin | Pogostol | PC8 |
| Pogostemon Cablin | Perilla Ketone | PC9 |
| Radix Curcumae | Zerumbone | RC1 |
| Radix Curcumae | naringenin | RC10 |
| Radix Curcumae | Isorhamnetin | RC13 |
| Radix Curcumae | Germacrene a | RC15 |
| Radix Curcumae | Ethyl ferulate | RC16 |
| Radix Curcumae | Demethoxycurcumin | RC17 |
| Radix Curcumae | Curlone | RC19 |
| Radix Curcumae | Zedoalactone A | RC2 |
| Radix Curcumae | Curdione | RC20 |
| Radix Curcumae | Curcumenolactone C | RC21 |
| Radix Curcumae | Curcumalactone | RC22 |
| Radix Curcumae | Curcarabranol B | RC23 |
| Radix Curcumae | cis-p-Coumaric acid | RC24 |
| Radix Curcumae | cis-Cinnamic acid | RC25 |
| Radix Curcumae | cis-Caffeic acid | RC26 |
| Radix Curcumae | Cirsilineol | RC27 |
| Radix Curcumae | Cibarian | RC29 |
| Radix Curcumae | Turricolol E | RC3 |
| Radix Curcumae | CaryoPCyllene | RC30 |
| Radix Curcumae | Capillarisin | RC31 |
| Radix Curcumae | CamPCerenol | RC33 |
| Radix Curcumae | Bisdemethoxycurcumin | RC35 |
| Radix Curcumae | Bicyclo(4.1.0)heptan-3-one, 4-(1-hydroxy-1-methylethyl)-1-methyl-7-(3-oxobutyl)-,(1S,4S,6R,7R)- | RC36 |
| Radix Curcumae | beta-sitosterol | RC37 |
| Radix Curcumae | 10-epi-gamma-Eudesmol | RC38 |
| Radix Curcumae | 1,7-DiPCenyl-4,6-heptadien-3-one | RC39 |
| Radix Curcumae | Tomatideno | RC4 |
| Radix Curcumae | (Z)-p-Methoxycinnamic acid | RC40 |
| Radix Curcumae | (Z)-caryoPCyllene | RC41 |
| Radix Curcumae | (E)-2-epi-beta-caryoPCyllene | RC43 |
| Radix Curcumae | (3R,10S)-6,10-dimethyl-3-propan-2-ylcyclodec-6-ene-1,4-dion | RC44 |
| Radix Curcumae | (2R)-5,7-dihydroxy-2-(4-hydroxyPCenyl)-2,3-dihydro-4H-chromen-4-one | RC45 |
| Radix Curcumae | (+)-Terpinen-4-ol | RC47 |
| Radix Curcumae | (-)-Limonene | RC49 |
| Radix Curcumae | sitosterol | RC5 |
| Radix Curcumae | Isorhamnetin | RC55 |
| Radix Curcumae | Procurcumenol | RC7 |
| Radix Curcumae | PCellatin | RC8 |
| Radix Curcumae | NCGC00385952-01_C15H26O_1,7-Dimethyl-7-(4-methyl-3-penten-1-yl)bicyclo[2.2.1]heptan-2-ol | RC9 |
